# Polypoid Melanoma: A Rare and Aggressive Variant

**DOI:** 10.7759/cureus.103896

**Published:** 2026-02-19

**Authors:** Raj Patel, Calista Persson, Sacha Saba, Paul Steele

**Affiliations:** 1 Department of Dermatology, HCA Healthcare/USF Morsani College of Medicine GME, Largo, USA; 2 Osteopathic Medical School, Nova Southeastern University Dr. Kiran C. Patel College of Osteopathic Medicine, Fort Lauderdale, USA

**Keywords:** aggressive melanoma, breslow thickness, diagnostic delay, exophytic cutaneous lesion, immunohistochemistry, immunotherapy, nodular melanoma, polypoid melanoma, rare melanoma, sentinel lymph node biopsy (slnb)

## Abstract

A 57-year-old man presented with a several-month history of a bleeding, lobulated mass on the left lower back and a palpable left axillary lymph node. Excisional biopsy revealed an 11-mm-thick, lobulated, sessile polypoid nodular melanoma (pT4b) with Clark level IV invasion, a mitotic rate of 4/mm², and associated ulceration. A sentinel lymph node biopsy was not performed, as the patient was subsequently lost to follow-up. Immunohistochemistry was positive for SOX10, MART-1, and HMB-45, confirming melanocytic differentiation. The lesion was initially excised with 2-cm clinical margins, and histopathologic evaluation confirmed negative margins. Although referral was made for wide local excision and sentinel lymph node biopsy for definitive staging and management, the patient did not return for additional surgical intervention and was lost to follow-up.

Polypoid melanoma (PM) typically presents as rapidly enlarging, exophytic, and often ulcerated or bleeding nodules that can mimic benign vascular or inflammatory lesions (e.g., pyogenic granuloma), which may delay diagnosis. Prompt recognition and early biopsy of atypical exophytic nodules are therefore essential. Management emphasizes timely wide excision and accurate pathologic staging, with multidisciplinary coordination and consideration of adjuvant systemic therapy (such as immune checkpoint inhibitors or targeted agents) when indicated by stage. This case highlights the need to maintain a high index of suspicion for PM in atypical, rapidly growing, or bleeding cutaneous nodules.

## Introduction

Polypoid melanoma (PM) is an unusual subtype of melanoma that accounts for a disproportionately high rate of morbidity and mortality [1]. It was initially described clinically in 1958, in a series of cases of malignant melanoma [2]. Since then, it has been recognized as a distinct clinicopathologic entity, with a presentation and disease course that differ substantially from other forms of melanoma. PM typically presents as a rapidly enlarging, cauliflower-shaped mass and may be complicated by ulceration or hemorrhage [3]. Its exophytic and sometimes friable morphology can lead clinicians to mistake it for non-melanocytic tumors, such as pyogenic granulomas or other vascular lesions, further contributing to diagnostic delays. It has been recently shown to be associated with aggressive histopathologic features and poor prognosis [4]. Due to its atypical presentation, PM is often misdiagnosed, leading to delayed diagnosis and consequently unfavorable outcomes [5]. We report a case of a patient with truncal PM, which further highlights its aggressive course and diagnostic challenges.

## Case presentation

A 57-year-old man presented to the emergency department with a rapidly enlarging bleeding nodule on the left lower back, first noticed several months prior. The lesion caused recurrent bleeding episodes that stained clothing and interfered with daily activities, significantly affecting his quality of life. He also noted pain and a new left axillary mass.

**Figure 1:**
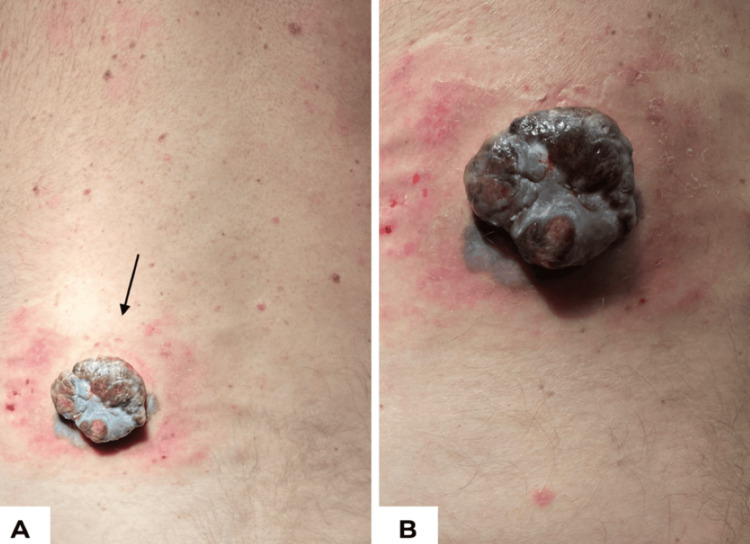
Clinical images of a 52-year-old man with polypoid melanoma. (A) Distant view of a 4.5 × 4.0 cm exophytic, ulcerated, pigmented nodule (arrow) on the left lower back. (B) Close-up view showing lobulated surface and surrounding erythema.

On thorough examination, there was a 4.5 × 4.0 cm exophytic, multi-pigmented, lobulated lesion with ulceration, hemorrhage, fibrinous tissue, peripheral erythema, and irregular pigmentation extending around the base of the nodule (Figure 1). Dermoscopy revealed marked asymmetry with a predominantly structureless pattern of irregular blue-black pigmentation, a polymorphous vascular pattern (dotted vessels and milky-red areas), focal ulceration and hemorrhage, and a blue-white veil. A palpable ipsilateral level I axillary lymph node was detected on physical examination, concerning for regional metastasis. Further nodal evaluation, including sentinel or non-sentinel lymph node biopsy, was not performed because the patient was lost to follow-up.

Excisional biopsy demonstrated an 11-mm Breslow thickness [6], classified as pT4b per American Joint Committee on Cancer (AJCC) criteria [7], consistent with a sessile polypoid nodular melanoma exhibiting Clark level IV invasion and a mitotic rate of 4/mm² [8]. Comprehensive staging, including cross-sectional imaging (CT/PET) and sentinel lymph node biopsy, was recommended; however, these evaluations were not performed, as the patient was lost to follow-up, precluding definitive regional or distant staging.

**Figure 2:**
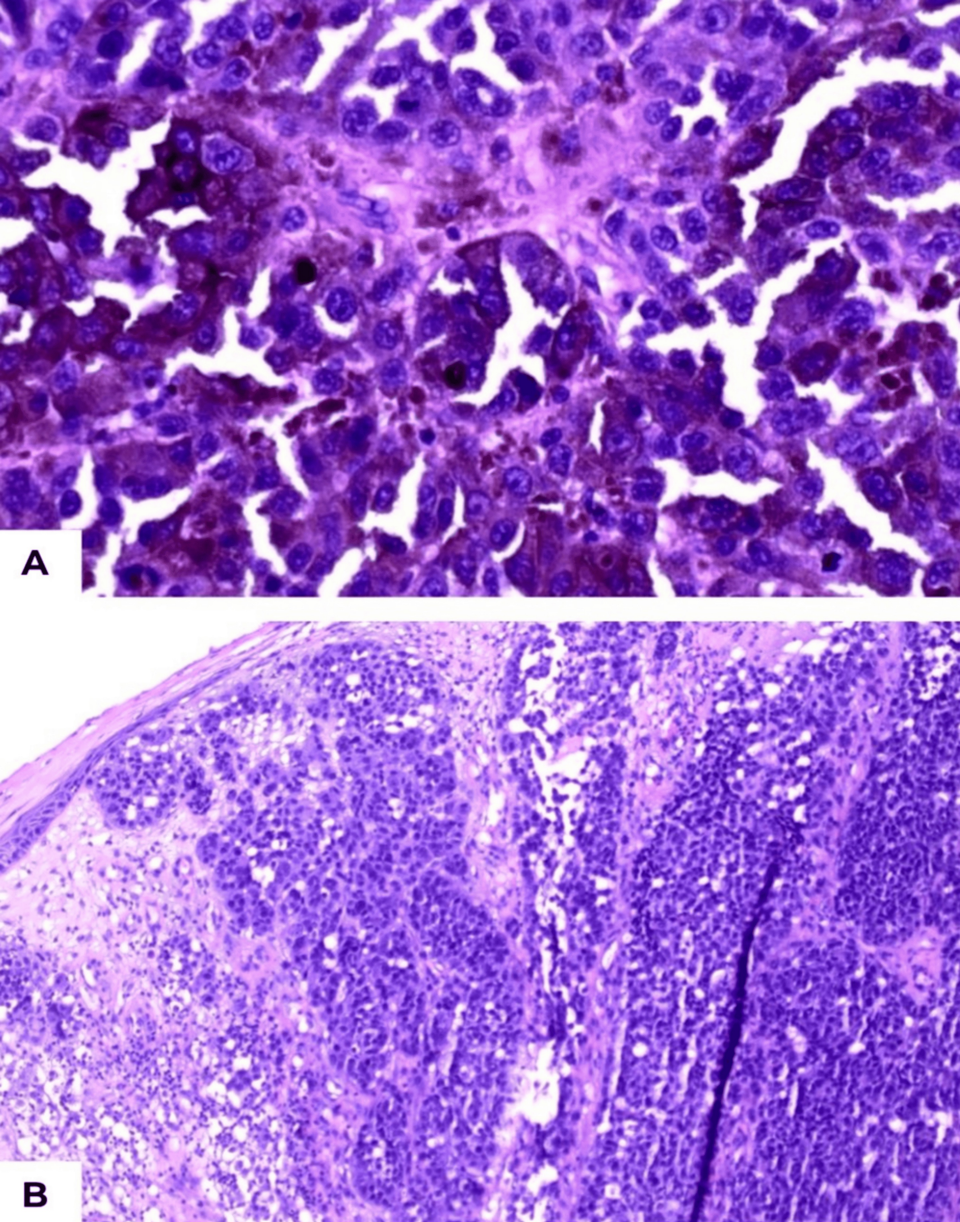
Histopathology of polypoid melanoma on hematoxylin and eosin stain. (A) Low-power view (×100) showing a nodular proliferation of atypical melanocytes infiltrating the dermis. (B) High-power view (×400) highlighting marked nuclear pleomorphism, frequent mitoses, tumor-infiltrating lymphocytes, and scattered melanophages. Immunohistochemistry performed on parallel sections demonstrated diffuse nuclear SOX10 positivity and cytoplasmic/membranous positivity for MART-1 and HMB-45, supporting melanocytic differentiation [9].

Histopathology showed nodular proliferation of atypical melanocytes with tumor-infiltrating lymphocytes and melanophages. Immunohistochemical stains were positive for SOX10, MART-1, HMB-45, CK7, CK20, CD45, and vimentin (Figure 2) [9]. The primary lesion was excised with 2 cm clinical surgical margins, consistent with oncologic safety principles for widely excising aggressive cutaneous lesions. Permanent section analysis confirmed histologically negative margins, indicating complete excision of the tumor with no residual disease at the resection edges, as described in prior reports where 2-cm clinical margins yielded negative histopathologic margins [10]. These findings, in conjunction with the clinical presentation, confirmed the diagnosis of polypoid melanoma. Referral was made for wide local excision and sentinel lymph node biopsy to complete definitive surgical management and staging. Unfortunately, the patient did not present for further evaluation, and these procedures were not performed due to loss to follow-up.

## Discussion

PM, a locally aggressive subtype of melanoma, is characterized by thicker lesions (mean Breslow’s thickness of 5.9 mm), higher mitotic rates, and a greater incidence of ulceration. These features not only distinguish PM from conventional nodular melanoma but also help explain its rapid progression and poor prognosis. Compared with conventional nodular melanoma, PM tends to present at more advanced stages, with studies showing significantly poorer overall survival rates [3]. Its cauliflower-like, exophytic morphology often resembles benign conditions such as pyogenic granulomas or seborrheic keratoses, and irregular pigmentation may further obscure its malignant potential. As a result, diagnosis is frequently delayed, and many patients present with thick primaries or nodal disease [2,4].

Histologically, PM demonstrates pronounced cytologic atypia, marked nuclear pleomorphism, and brisk vertical growth, features that promote early vascular invasion and metastatic dissemination [4]. The dermal component is typically bulky and disproportionately expanded relative to the epidermal component, often accompanied by frequent mitoses, tumor-infiltrating lymphocytes, and melanophages [3]. The transition from radial to vertical growth occurs abruptly, which likely contributes to its rapid clinical progression. This early vertical dominance facilitates prompt access to lymphatic and vascular channels, accounting for the aggressive biologic behavior observed in this subtype [11,12].

Prognosis in PM is largely driven by established melanoma prognostic markers, particularly Breslow thickness, ulceration status, and nodal involvement. Increasing tumor thickness remains the most significant predictor of melanoma-specific survival, with lesions greater than 4 mm (T4) demonstrating substantially worse outcomes compared to thinner tumors [7,13]. Ulceration further independently worsens prognosis and results inupstaging within the AJCC classification system, correlating with reduced survival across T categories [7,14]. Regional lymph node metastasis represents another critical determinant of outcome, as sentinel lymph node positivity significantly decreases disease-free and overall survival [15]. Historically, PM has been associated with higher mortality than other melanoma subtypes [1], a finding that persists in contemporary analyses and is largely attributable to its greater thickness at presentation and aggressive vertical growth pattern [3].

In addition to its aggressive clinical course, PM demonstrates characteristic histopathologic and immunophenotypic features that may aid in distinguishing it from other nodular melanomas. It is typically marked by a disproportionately bulky dermal component, exophytic architecture, and frequent ulceration, often presenting with significant tumor thickness at diagnosis [2,4,5]. Immunohistochemically, PM retains expression of melanocytic markers including S100, SOX10, HMB-45, and Melan-A, which confirm melanocytic differentiation and help exclude poorly differentiated non-melanocytic malignancies in clinically ambiguous cases [9]. Integration of these morphologic findings with standardized staging criteria, including nodal assessment when available, remains essential for accurate classification and prognostic stratification [7,13,15]. Contemporary reports emphasize that delayed recognition contributes substantially to advanced-stage presentation, underscoring the importance of early identification of this distinctive variant [11,12,14].

In this case, the patient underwent excisional biopsy with 2-cm clinical margins, which were histologically negative. Referral was made for wide local excision and sentinel lymph node biopsy to complete definitive staging and management in accordance with established melanoma guidelines [7,13]. However, the patient did not return for further treatment and was subsequently lost to follow-up, precluding assessment of short- or long-term outcomes.

Wide local excision remains the standard treatment for primary melanoma, with margin recommendations guided by Breslow thickness and AJCC staging criteria [6,7,13]. In many cases, management of bulky polypoid lesions on the trunk or extremities necessitates complex closures, including skin grafting or flap reconstruction, to achieve recommended 2-cm margins when anatomically feasible. Truncal excisions in particular may present functional and cosmetic challenges, as surgeons must balance oncologic clearance with preservation of surrounding structures. Recent surgical innovations have facilitated wider resections of large trunk lesions while improving oncologic control, and adjunctive techniques such as staged excisions and intraoperative frozen-section margin assessment may further optimize margin evaluation in select cases [10]. Sentinel lymph node biopsy remains an important staging tool in clinically node-negative patients and provides significant prognostic information when performed [7,15].

Systemic therapy has become an increasingly critical component in the management of advanced melanoma. Immune checkpoint inhibitors, including nivolumab, have demonstrated durable responses even in cases of delayed diagnosis, and combination regimens such as nivolumab with ipilimumab have shown improved response rates, albeit with higher rates of immune-related adverse events [11]. For tumors harboring actionable mutations, targeted therapy with BRAF and MEK inhibitors offers an additional effective strategy. Emerging neoadjuvant immunotherapy trials suggest that earlier systemic intervention may downstage bulky tumors and potentially enhance surgical outcomes. These advances provide cautious optimism that even historically aggressive variants such as PM may benefit from modern multimodal treatment approaches [11].

PM is associated with high recurrence and melanoma-specific mortality rates [1,2]. In the present case, the patient underwent excisional biopsy with 2-cm histologically negative margins; however, he did not proceed with the recommended wide local excision or sentinel lymph node biopsy and was subsequently lost to follow-up. Consequently, definitive pathologic staging and long-term outcomes could not be assessed. Given the presence of high-risk features, including an 11-mm Breslow thickness, ulceration, and clinically palpable regional lymphadenopathy-completion wide local excision with sentinel lymph node biopsy would have been strongly indicated in accordance with current AJCC guidelines [7,13,15], with consideration of adjuvant systemic therapy in the setting of node-positive or advanced disease [11].

Due to its aggressive biologic behavior, management of PM extends beyond definitive excision. For patients with similarly high-risk features, close surveillance is recommended, including comprehensive skin and regional nodal examinations every three to six months during the first several years, with consideration of cross-sectional imaging to facilitate early detection of recurrence [7,13]. Early initiation of adjuvant immunotherapy in appropriate candidates may further improve recurrence-free survival [11,15]. Collectively, these measures underscore that prognosis in PM depends not only on timely diagnosis and adequate surgical management but also on structured follow-up and coordinated multidisciplinary care.

## Conclusions

PM, a rare yet highly aggressive variant, underscores the critical importance of early recognition and intervention. Its exophytic, bleeding morphology should immediately raise suspicion and prompt biopsy. The atypical appearance of PM contributes to frequent misdiagnosis, often resulting in an advanced stage at presentation. Definitive management rests on urgent biopsy, wide surgical excision, and the comprehensive approach of integrating systemic therapy when indicated. This comprehensive approach to treatment is crucial in managing this uncommon but formidable melanoma subtype. Raising awareness among clinicians about PM may shorten the time to diagnosis, reduce morbidity, and ultimately improve survival for patients.
